# Effect of a Six-Month Dance Intervention on Postural Control and Fall-Related Outcomes in Older Adults with Mild Cognitive Impairment: A Randomized Controlled Trial

**DOI:** 10.3390/geriatrics10030067

**Published:** 2025-05-17

**Authors:** Ulrich Thiel, Nicole Halfpaap, Berit K. Labott, Fabian Herold, Corinna Langhans, Kristinn Heinrichs, Patrick Müller, Notger G. Müller, Anita Hökelmann

**Affiliations:** 1 Department of Sport Science, Faculty of Humanities, Otto-von-Guericke University Magdeburg, 39104 Magdeburg, Germany; 2 Department of Neurology, Medical Faculty, Otto-von-Guericke University Magdeburg, 39120 Magdeburg, Germany; 3 Department of Neuromotor Behavior and Exercise, University of Münster, 48149 Münster, Germany; 4 Research Group Degenerative and Chronic Diseases, Movement, Faculty of Health Sciences Brandenburg, University of Potsdam, 14476 Potsdam, Germany; 5 Research Group Neuroprotection, German Center for Neurodegenerative Diseases (DZNE), 39120 Magdeburg, Germany; 6 Department of Physiology, Faculty of Medicine, HMU Health and Medical University Erfurt, 99084 Erfurt, Germany; 7 Bertec Corporation, Columbus, OH 43219, USA; 8 Department of Cardiology and Angiology, Medical Faculty, Otto-von-Guericke University Magdeburg, 39120 Magdeburg, Germany; 9 Centre for Intervention and Research on Adaptive and Maladaptive Brain Circuits Underlying Mental Health (C-I-R-C), 39120 Magdeburg, Germany; 10 German Centre for Mental Health (DZPG), 39120 Magdeburg, Germany; 11 German Centre for Neurodegenerative Diseases (DZNE), 39120 Magdeburg, Germany

**Keywords:** exercise therapy, postural balance, rehabilitation, aging, cognitive dysfunction

## Abstract

Background/Objectives: Older adults with mild cognitive impairment often exhibit reduced postural control and increased fall risk. As fall-related injuries consume substantial healthcare resources, the development of fall-preventive interventions is of public health relevance. This study aims to investigate the effects of a six-month dance intervention on postural control and fall-related measures in older adults with mild cognitive impairment. Methods: In this randomized controlled trial, 55 participants were allocated to either an intervention group or a control group. The intervention group performed two 90-min dance training sessions per week for six months, while the control group maintained their usual activities of daily living. Postural control was operationalized via balance performance, which was measured with the Sensory Organization Test and the Limits of Stability Test. Neuromuscular function of the lower extremities was assessed via muscle contraction velocity using tensiomyography. Fear of falling was quantified with the Falls Efficacy Scale, and participants reported fall history over the past year. It was hypothesized that older adults with mild cognitive impairment participating in the six-month dance training would show significantly greater improvements in postural control and fall-related outcomes than those in the control group. Results: A mixed analysis of variance (time × group) revealed no significant improvements in balance performance or neuromuscular function following the dance intervention (*p* > 0.05). However, several main effects for time were observed in the Sensory Organization Test, Limits of Stability Test, and muscle contraction velocity. Scores on the Falls Efficacy Scale improved significantly in the intervention group, reflecting reduced fear of falling, although only shown by a paired-samples *t*-test (t(23)= 2.276, *p* = 0.032, d = 0.465). Conclusions: This study did not provide evidence that a six-month dance intervention improves postural or neuromuscular functions. However, it cannot be ruled out that such null findings are related to confounding factors, such as insufficient training specificity or duration. Nonetheless, the fear of falling was significantly reduced in the intervention group, suggesting potential benefits for perceived fall risk in older adults with mild cognitive impairment.

## 1. Introduction

The preservation of postural control is a key aspect of reducing the risk of falling (ROF) in older adults [1]. Typically, the ROF increases with age due to alterations in neuromuscular and sensory systems [2,3,4]. These alterations, which are related to structural and functional changes, contribute to lower postural control and include, but are not limited to, decreased muscle mass and strength, slower reaction time (RT), and a deterioration of neuromuscular coordination and sensory integration [1,5,6,7,8,9]. Sensory integration is an important process for ensuring the well-functioning of postural control, and age-related sensory changes to proprioception, vision, or the vestibular system increase difficulties in maintaining appropriate postural control, such as the reaction to sudden changes in balance [10,11]. Therefore, age-related changes in those factors may impair postural control and increase the ROF in the general population of older adults. In this study, a Sensory Organization Test (SOT) is used to investigate the participant’s ability to integrate sensory information to maintain postural control.

Intra- and intermuscular coordination is essential to ensure balance in case of rapid changes in center of pressure, defined as the theoretical vector on which the total force acts on the person’s feet if it were concentrated in one spot [12]. Typically, older adults react more slowly to disturbances in the center of pressure, which is, among other factors, related to a deterioration in neuromuscular function [5,6]. Such neuromuscular deficits can significantly increase the ROF, as the efficiency of postural control is affected by age-related changes in the sensory and motor systems [9,13]. We used the Limits of Stability Test (LOS) to assess the participant’s ability to maintain balance while leaning in different directions and tensiomyography to investigate the neuromuscular functioning responsible for movement control, in particular the muscle contraction velocity (Vc).

Recent studies show that physical activity, especially planned and structured forms referred to as physical exercise [14], can promote physical health and cognitive abilities, specifically in the areas of memory and executive function [15,16]. This is particularly relevant for older adults with mild cognitive impairment (MCI), which is described as a transitional stage between healthy aging and dementia [17], since motor and cognitive impairments occur simultaneously and often reinforce each other [18]. Thus, MCI has been shown to negatively affect specific measures of postural control [7]. However, improving physical and cognitive abilities contributes to maintaining independence and improving quality of life in older age. The ability to cope independently with everyday life is closely linked to mental and physical health; physical activity can significantly improve well-being in older adults [19,20]. The loss of mobility and independence increases the risk of social isolation and depression, further accelerating cognitive decline in a vicious cycle [19]. Planned and structured forms of physical activity may additionally reduce the ROF by enhancing neuromuscular (e.g., muscle contractile properties) and cognitive performance (e.g., working memory) to effectively complete complex movement tasks occurring during daily living [21,22]. To investigate the ROF, we measured the participant’s fear of falling using the Falls Efficacy Scale–International (FES-I) as well as their recent number of falls.

Studies have demonstrated that motor training combined with cognitive challenges has positive effects on specific cognitive functions (i.e., executive function). Preserving cognition could help to ensure the independence of community-dwelling older adults and reduce or delay the need for assisted living or dependence [15,23].

As older adults are most at risk during daily life activities—such as walking, turning, and transitioning between positions—interventions, such as functional training, that simulate these tasks are likely to translate into real-world benefits [24,25]. Functional training approaches have also been shown to enhance motivation and adherence, particularly when they are socially engaging and embedded in meaningful activities [26]. In this context, dance-based interventions may offer a promising solution, as they integrate dynamic balance challenges, rhythm, motor coordination, and social interaction within a functionally relevant framework.

Dance training, as a multimodal intervention, can be individualized to account for the different baseline fitness levels of trainees. For example, the tempo of the music can be either slow or fast, resulting in lower or higher exercise intensity, respectively. An increased number of turning movements required in a specific type of dancing may increase the coordinative demands by posing a higher load on the visual–vestibular system. Taken together, dance training, which primarily involves choreographed rhythmic movements and thus challenges different abilities such as coordination, spatial navigation, working memory, and spatial memory, is a promising intervention approach to improve postural control and cognitive functioning, thus also resulting in a lower fall risk [27,28]. Indeed, several studies observed that dance movements can improve balance and motor control and strengthen memory and executive functions [27,29]. In addition, dance as a social activity creates a motivating framework, which may lead to a higher adherence to the training intervention [23]. However, whether such positive effects seen in healthy older adults generalize to older adults with MCI is relatively poorly understood.

Thus, this study investigated the effects of a six-month dance intervention on postural control—operationalized by balance and neuromuscular performance (specifically, Vc of the lower extremities)—and fall risk, measured by fear of falling and the number of falls, in older adults with MCI.

## 2. Materials and Methods

### 2.1. Participants

As part of the “DiADEM—Dance Against Dementia” research project, older adults with mild cognitive impairment (MCI) were recruited from advertisements in local newspapers, flyers, notices, word of mouth, and existing databases. During the recruitment process, interested individuals were screened for eligibility using the following inclusion criteria: (i) age between 50 and 80 years, (ii) German as the first and primary language, and (iii) ability to manage daily activities independently.

Exclusion criteria included the following: (a) severely impaired or uncorrected vision or hearing, color vision deficiency or color blindness; (b) severe psychiatric illness (e.g., bipolar disorder) or depression (assessed using the Geriatric Depression Scale; cut-off value ≥ 6 [30]); (c) orthopedic injury or surgery (e.g., fracture or surgery in the last six months and symptomatic intervertebral disk pathologies); (d) severe muscular illness (e.g., myositis and tendovaginitis); (e) severe cardiovascular diseases (e.g., heart failure); (f) endocrinological diseases (e.g., manifest hypothyroidism or hyperthyroidism, insulin-dependent diabetes mellitus type I and II, and BMI > 30); (g) neurological diseases other than MCI (e.g., stroke, epilepsy, and multiple sclerosis); (h) major injuries or operations in the last six months; and/or (i) the use of certain medications (e.g., neuroleptics, narcotic analgesics, benzodiazepines, and psychoactive medications) and/or the use of illegal substances or alcohol abuse. Pregnant women were also excluded from participation.

Participants with MCI were identified according to the criteria of Petersen (2004), who defined MCI as cognitive performance below the age-appropriate level without symptoms of manifest dementia (DSM-IV and ICD-10) [31]. Individuals with MCI showed (i) maintenance of basic activities of daily living and only minor limitations in complex instrumental functions; (ii) self-reported or family-described cognitive impairment; and/or (iii) evidence of decline in objective cognitive tasks over time. The Consortium to Establish a Registry for Alzheimer’s Disease Plus test battery was used to check these criteria, whereby a performance below −1.5 SD in the age-, gender-, and education-adjusted z-score was interpreted as MCI in at least one subtest. A minimum score of at least 24 in the Mini-Mental State Examination excluded cases of dementia [32]. The participants were further divided into two subtypes of MCI, amnestic and non-amnestic MCI. This classification was created based on the performance in the delayed recall trial of the Wordlist and Figure episodic memory test (included in the Consortium to Establish a Registry for Alzheimer’s Disease Plus test battery) [33]. Participants were classified as amnestic MCI with a performance below −1.5 SD in the age, sex, and education-adjusted z-value in at least one of the memory tests, and those with a score higher than −1.5 SD were classified as non-amnestic MCI [33]. The diagnosis of MCI was then verified by an experienced neurologist who performed a standard neurological examination.

**Figure 1:**
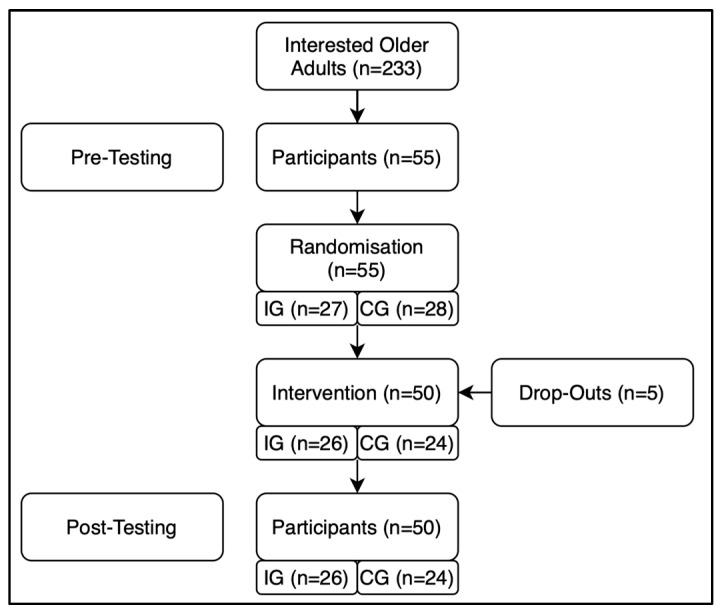
Study design flowchart.

The required sample size was calculated a priori with G*Power Version 3.1 [34]. With an effect size of Cohen’s f = 0.26 (medium) [35], 52 participants were needed to achieve a statistical power of 0.96 in regard to a repeated measure analysis of variance (within–between interaction). Of all interested older adults (n = 233), 55 met the eligibility criteria for participation in this study. Random allocation of the 55 participants was performed using stratified randomization based on age and sex via the software offered on the following website: https://www.ultimatesolver.com/en (accessed on 1 March 2020).Recruitment and randomization were carried out by two separate members of the research team to prevent bias. The allocation sequence was concealed from the enrolling personnel and participants until group assignment into either the intervention group (IG) or the control group (CG) was completed. Afterwards, one couple was reassigned within the groups to ensure that they were allocated to the same group. All participants were asked to maintain their usual lifestyle, while the intervention group also participated in dance training twice weekly. Only participants attending at least 80% of the dance training sessions were included in the analysis. The study design is shown in Figure 1.

Before the start of this study, all participants were informed of the study design and the possible risks and benefits and gave their written consent to participate. The intervention was conducted in a sports hall at the Otto-von-Guericke University Magdeburg, while data collection took place in adjacent laboratories.

The study procedures complied with the ethical guidelines of the current version of the Declaration of Helsinki and were approved by the Ethics Committee of the Medical Faculty of the Otto-von-Guericke University Magdeburg (reference number: 17/20). This study was pre-registered in the German Clinical Trials Register [DRKS00022575] and is reported in accordance with the CONSORT 2010 guidelines for randomized controlled trials [36].

### 2.2. Intervention

During the six-month intervention, the IG completed twice-weekly 90-min dance sessions on non-consecutive days (i.e., on Wednesdays and Fridays). Each 90-min session was divided into the following three sections: a 15-min warm-up, a 60-min main part divided into two segments (coordination exercises followed by strength/endurance exercises), and a 15-min cooldown.

Led by a qualified instructor with experience in dance programs for older adults, the dance training aimed to improve coordination and cognition, particularly memory performance. To this end, the participants continuously learned new dance choreographies, whereby individual movements were practiced in advance, then combined into individual sequences and later into an overall choreography to facilitate memorization. The dance genres included line dancing, jazz, square dancing, and Latin American styles, with a gradual increase in the music’s tempo to incrementally increase the speed and range of movement and exercise intensity. The line dance requires spatial orientation and navigation skills, motor planning, and simultaneous auditory and visual processing while moving in a dynamic and constantly changing environment. During the two dance parts of a single training session, two different dances were taught and practiced. The first dance of a training session focused on arm–leg coordination, while the second part aimed at improving muscular strength and endurance (e.g., using handheld weights, resistance bands, step boards, etc.).

The exercise intensity was controlled via a portable heart rate monitoring system (Polar Team Pro, Polar Electro GmbH, Büttelborn, Germany), with participants maintaining an exercise intensity in the range of 50–70% of their maximum heart rate, corresponding to a moderate to vigorous exercise intensity [37]. Each participant wore a sensor and chest strap (Polar Pro Sensor, Polar Electro GmbH, Büttelborn, Germany), transmitting real-time feedback to a tablet, allowing the exercise instructor to oversee exercise intensity levels through a color-coded display. A one-minute baseline resting phase before each session provided reference values.

The CG received no intervention and were asked to continue with their usual activities of daily living, including the maintenance of their regular physical activity level. As compensation, the CG was offered a structured dance program at a local dance club after this study was completed.

### 2.3. Balance Measurements

Computerized dynamic posturography is an established test able to measure both sensory and motor (automatic and voluntary) contributions to postural control [38], including but not limited to assessments such as the SOT and the voluntary goal-directed LOS, respectively. This assessment of sensory integration and postural control allows for an objective quantification of age-related changes in postural control strategies of older adults [39]. For example, composite SOT scores less than 38 have been associated with increased ROF, while a prolonged RT and a decreased movement velocity (MVL) on the LOS were found to be predictive of falling within 6 months [39]. In the current study, the Neurocom^®^ Balance Master (Natus Medical Incorporated, Middleton, USA) was used to collect SOT and LOS data.

#### 2.3.1. Sensory Organization Test

The SOT quantifies the contribution of multisensory integration (i.e., from the somatosensory, visual, and vestibular systems) to maintain postural control while standing on a dynamic balance plate [40]. This allows us to isolate and quantify the ability to utilize a specific sensory system by systematically manipulating the visual input and balance platform environments relative to baseline conditions. SOT measures both the sway characteristics of the center of pressure (amplitude, direction, frequency) and the maximum anterior and posterior center of gravity (COG) displacement relative to the theoretical limits of stability.

**Figure 2:**
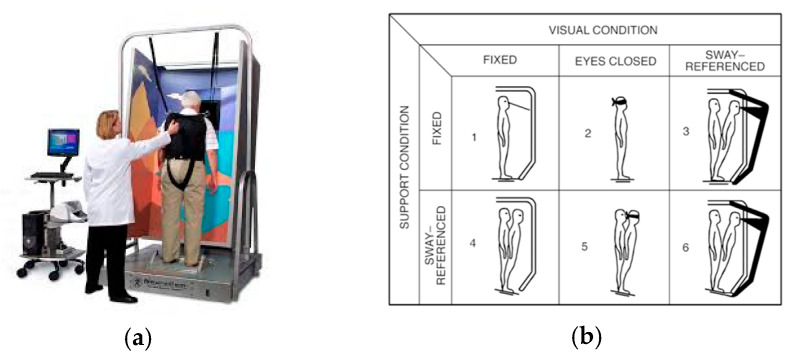
(**a**) Balance master (left side) [42]. (**b**) SOT conditions (right side) (1–3: stable platform, somatosensory dominant; conditions 4–6: sway-referenced platform absent somatosensory information; conditions 3 and 6: sway-referenced vision with inaccurate visual cues; conditions 2 and 5: absent visual information; condition 4: vision; condition 5: pure vestibular; and condition 6: secondary vestibular) [43].

The system calculates equilibrium scores for six conditions (Figure 2b). These equilibrium scores reflect the participants’ sway amplitude, where a value of 100 represents no sway and a value of 0 determines a complete loss of balance [41]. Additionally, a composite score is calculated as a weighted average of the six conditions (three trials per condition).

In addition, the SOT yields information to the extent that an individual utilizes visual and vestibular information even when that information is incorrect. Eq-1 is the reference condition for the SOT and denominator for the sensory score ratios, as it reflects static balance under normal conditions (eyes open, stable floor). The somatosensory system is the dominant system when the floor is stable. To remove somatosensory input, sway-referencing of the floor must occur. Therefore, vision is the dominant sensory system used in condition 4. The somatosensory score is calculated as Eq-2/Eq-1, the vision score was calculated as Eq-4/Eq-1, and the vestibular score was calculated as Eq-5/Eq-1. The visual preference sensory score ratio was calculated as Eq-3 + Eq-6 (inaccurate visual cues)/Eq-2 + Eq-5 (no visual cues) and is a measure of the extent to which an individual uses visual information to maintain balance even when this visual information is incorrect.

#### 2.3.2. Limits of Stability

**Figure 3:**
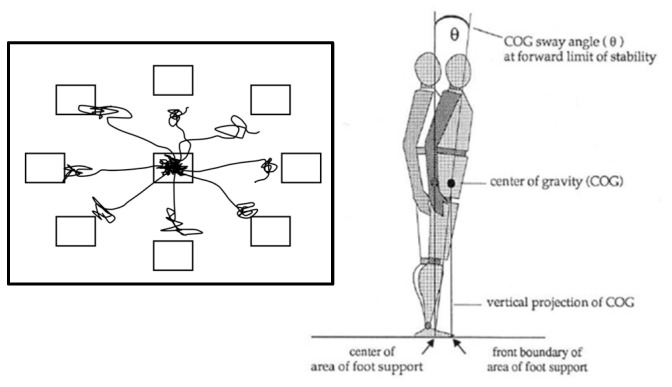
Starting positions of the LOS. Lines on the diagram represent the target movement paths and the actual movement pattern of the subject during the test (**left**). The image on the right explains the biomechanical principles behind the limits of stability. It shows the center of gravity (COG), COG sway angle (θ), vertical projection of the COG, and movement limits [46].

The LOS quantifies how closely an individual can approach the theoretical limit of stability and maintain balance without a stepping response. The functional LOS is the maximum distance measured as the COG sway angle from vertical that an individual can lean their COG without losing balance or initiating a “recovery” step [44]. Upon seeing a “go” signal on a screen in front, the participants were instructed to move a cursor by leaning toward one of eight targets as quickly, accurately, and as far as possible and to hold the position until signaled to return to the start position. The eight targets are in anterior, posterior, and mediolateral directions (see Figure 3). Therefore, it is a voluntary motor control test used to determine the extent to which an individual can quickly and accurately shift their body weight to move a cursor toward each of the eight targets in turn [45]. Healthy individuals (e.g., with a normal range of motion and muscular strength) can achieve sway angles of 8.0 degrees forward, right, and left, and 4.5 degrees backward (Figure 3). When the COG exceeds the base of support, the individual must initiate a “recovery” step to avoid stumbling or falling.

Four trials corresponding to the following directions were selected for analysis: forward, right, backward, and left. Based on these trials, the following five parameters were calculated: (i) RT: the time in seconds from the “go” to the start of the movement of the center of gravity from the rest position; (ii) MVL of the COG (°/s); (iii) maximum excursion (MXE) or the farthest displacement the COG reaches toward the target on the first attempt to lean toward the target; (iv) endpoint excursion (EPE), measuring the participants’ self-limitations due to fear of falling; and (v) directional control (DCL) as a measure of movement quality by comparing the amount of movement in the intended direction toward the target to the amount of extraneous movement away from the target. EPE, MXE, and DCL are reported as a percentage of the theoretical maximum limit of stability.

### 2.4. Muscle Contraction Velocity

Tensiomyography is a non-invasive method for analyzing the contractile properties of a muscle triggered by electrical stimulation. Using a contact displacement sensor, the radial displacement of the muscle belly is measured during an induced muscle twitch, providing information about muscle contraction and function [47]. Tensiomyography was used to measure the increase in muscle belly thickness in a transverse plane during an isometric muscle twitch contraction [48]. A high-precision digital displacement sensor (digital optical comparator, TMG-BMC Ltd., Ljubljana, Slovenia), which was pressed onto the muscle belly during the measurement using a spring (0.2 N/cm^2^), was used to ensure a high signal-to-noise ratio and high reliability [49]. The sensor was positioned perpendicular to the tangential plane on the skin over the muscle belly. All measurements were taken isometrically in relaxed, predetermined positions for the three muscles of the lower extremities as follows: (i) for the m. rectus femoris and the m. tibialis anterior in the supine position with a knee angle of 30° (where 0° represents a fully extended joint position); (ii) for the m. tibialis anterior in a position in which the ankle was in a neutral position; and (iii) for the m. semitendinosus in the prone position with a knee angle of 5°. Foam pads were used to stabilize the joints. If necessary, the measuring point and electrode positions were adjusted to achieve maximum muscle belly diameter. Using an electric stimulator (TMG-S2, TMG-BMC Ltd., Ljubljana, Slovenia), a rectangular pulse (twitch) of 1 ms was applied via stimulation electrodes positioned 5 cm distally (cathode) and 5 cm proximally (anode) from the measuring point. Initially, the current amplitude was set just above the threshold and then increased stepwise until the maximum radial displacement remained stable. The contractile properties of the muscle were calculated from two maximal twitch responses, with the average value being used for further analysis. Important tensiomyographic parameters include the maximum radial displacement, the delay time, the contraction time, the holding time, and the relaxation time [50].

The Vc was calculated based on the measurements of the maximum radial displacement and the contraction time and represents the speed at which the muscle reaches its maximum radial displacement. Examining the contraction time is particularly valuable as it allows for conclusions concerning the muscle’s ability to adapt to physical stress and thus serves as a reliable marker for changes in muscle condition [51]. Compared to the separate analysis of the maximum radial displacement and the contraction time, the analysis of Vc also has methodological advantages. Since the two values are closely related, an isolated interpretation of the contraction time without considering the maximum radial displacement can lead to erroneous conclusions [52]. Determining the Vc, which integrates both parameters, allows for a more comprehensive assessment of muscle status (e.g., level of fatigue) and adaptation [53,54].

### 2.5. Falls Efficacy Scale

The German version of the FES-I consists of 16 items and assesses the “fear of falling” as well as the associated self-efficacy of the participant with a very high reliability [55]. The participants were asked to score their concerns about falling during different activities of daily living (e.g., dressing, walking on uneven terrain) on a 4-point Likert scale with 0 as an anchor for not concerned at all and 4 as an anchor for very concerned [56]. A sum of points, ranging from 16 (no fear) to 64 (strong fear) and labeled as “score”, was determined as a proxy for the fear of falling. In addition to the FES-I, the participants were also asked about the number of falls in the last 12 months.

### 2.6. Statistical Analysis

Statistical analysis was performed using SPSS (IBM SPSS Statistics for Windows, Version 28.0. Armonk, NY, USA: IBM Corp.). The significance level of all tests was set to α = 0.05. First, descriptive statistics were calculated, including mean values, standard deviations, and 95% confidence intervals. Outliers were detected via boxplot diagrams, checked for likelihood of occurrence by deviation, and dismissed when the mean was outside of three standard deviations. Normal distribution was assessed via the Shapiro–Wilk test and visual inspection of the Q-Q plots.

Inferential statistics were performed using a mixed-design analysis of variance (ANOVA) with a 2 × 2 configuration with time (pre, post) as the within-subjects factor and group (control, intervention) as the between-subjects factor. Levene’s test was used to assess the homogeneity of error variances. The FES-I did not show homogeneous error variances; thus, no interaction effects were calculated. Instead, paired-samples and unpaired-samples *t*-tests were used for both groups. To control for the risk of false positives due to multiple comparisons, the false discovery rate was controlled using the Benjamini–Hochberg procedure with a threshold of Q = 0.05 [57]. Effect size for the ANOVA was calculated as η^2^_p_ and interpreted as follows: ≥0.01 to <0.06: small effect; ≥0.06 to <0.14: medium effect; ≥0.14: large effect [58]. Effect size for the *t*-tests was calculated as Cohen’s d, with values above 0.2 being interpreted as a small effect, above 0.5 as a medium effect, and above 0.8 as a strong effect [35].

## 3. Results

Out of fifty-five participants, five dropped out of this study during the intervention period, either due to non-intervention-related back pain (n = 1), cardiac problems (n = 1), or lack of time or lost interest (n = 3). The remaining participants maintained an attendance rate exceeding 80% throughout the intervention period. A total of 50 participants were included in the final statistical analyses (i.e., 26 participants of the IG and 24 of the CG). The general characteristics of the participants are listed in Table 1. Four participants were excluded from the tensiomyographic measurements due to personal decisions to withdraw. These participants either experienced heightened anxiety about the electrical stimulation or hypersensitivity regarding the procedure, which could not be mitigated during the preparation phase. A comparison of general characteristics between participants who completed the tensiomyographic measurements and those who did not reveal any significant differences (*p* > 0.05), suggesting that selection bias is unlikely. Furthermore, the data of seven participants was not included in the final statistical analysis of balance performance because of technical issues with the Balance Master system or difficulties in maintaining the required testing posture. Dropouts were deleted listwise, and pairwise deletion was used for participants with missing datasets. No imputation was performed. A post hoc power analysis using G*Power Version 3.1 with an effect size of Cohen’s f = 0.26 (medium) and the minimum sample size of 43 participants indicated a statistical power of 0.91 [34]. No intervention-related falls or other adverse events occurred during the study period.

### 3.1. Sensory Organization Test

Regarding the SOT, no statistically significant interaction effect between time and group and no significant main effect for group was observed (Table 2). A significant main effect on time was noticed regarding the visual system score (F = 12.445, *p* = 0.001, η^2^_p_ = 0.242 [large]).

### 3.2. Limits of Stability

After false discovery rate correction, no statistically significant interaction effects between time and group remained (Table 3). No significant main effect for groups emerged. Instead, significant main effects for time were found for four parameters: Forward MVL (F = 8.736, *p* = 0.006, η^2^_p_ = 0.2 [large]), Forward MXE (F = 8.669, *p* = 0.006, η^2^_p_ = 0.199 [large]), Right MVL (F = 5.489, *p* = 0.025, η^2^_p_ = 0.136 [medium]), and Backward RT (F = 4.995, *p* = 0.032, η^2^_p_ = 0.125 [medium]).

### 3.3. Muscle Contraction Velocity

The analysis of the Vc showed no statistically significant interaction effect between time and group and no significant main effect for group (Table 4). A significant main effect for time was observed for the left side of the m. rectus femoris (F = 7.19, *p* = 0.01, η^2^_p_ = 0.143 [large]) as well as for the m. semitendinosus on both the left side (F = 19,712, *p* < 0.001, η^2^_p_ = 0.314 [large]) and the right side (F = 20.106, *p* < 0.001, η^2^_p_ = 0.319 [large]).

### 3.4. Fear of Falling and Number of Falls

A paired-samples *t*-test revealed a significant reduction in the total score between the pre-test and post-test in the intervention group (18.9 ± 2.5 to 18.3 ± 2.9; *p* = 0.032), corresponding to a small effect size of d = 0.465. We also observed a trend towards a reduction in the number of falls (0.4 ± 0.9 to 0.0 ± 0.2; *p* = 0.088), corresponding to a small effect size of d = 0.364. The control group displayed non-significant changes following the intervention (Table 5). No between-group differences were found before the intervention (score: t(25.78) = 0.696, *p* = 0.493, d = 0.233, and number of falls: t(20.57) = 0.597, *p* = 0.557, d = 0.208) or emerged after the intervention (score: t(25.87) = 1.10, *p* = 0.280, d = 0.369, and number of falls: t(17.69) = 1.36, *p* = 0.192, d = 0.487).

## 4. Discussion

In older age, well-preserved postural control is a key factor for safe ambulation. Impaired postural control, especially in older adults, often leads to falls, which can result in serious injuries, limited independence, and increased mortality in older adults [59,60]. Trombetti et al. [60] also point out that impaired muscle health, a critical determinant of postural control, not only increases the risk of functional limitations but can also lead to increased fear of falling. A more pronounced fear of falling can lead to a loss of confidence to move safely and thus needs to be considered a serious threat to the autonomy of an individual because one may limit engagement in specific activities of daily living (e.g., walking to the city to visit friends) that have been identified as important factors for healthy aging (e.g., social interaction).

In particular, individuals with MCI show an increased ROF because MCI is often associated with subtle sensory and motor deficits beyond that of cognitive impairments, which can also affect postural control. For example, a study by Montero-Odasso et al. [61] shows that older adults with MCI have altered gait parameters and a reduced ability to quickly adapt to unexpected disturbances, which increases their susceptibility to falls. Thus, older adults with MCI can be considered an at-risk population for which appropriate interventions need to be developed to preserve not only cognitive but also physical health (i.e., lowering the fall risk). In this context, several studies in healthy older adults showed that dance training not only reduces the risk of dementia, as it involves both physical activity and cognitive challenges due to elements such as memorizing step sequences, reacting to music, and promoting social interactions [62,63], but also positively influences motor control, including postural [62,64] and gait control [65,66]. Based on the positive effects of dancing in healthy adults, we hypothesized that similar positive effects on balance would occur in older adults with MCI.

In addition to promoting motor and cognitive abilities, dancing in a group can also support social interaction, which is particularly important for individuals with MCI. Social contacts can reduce loneliness and increase emotional well-being, which in turn indirectly supports cognitive and motor performance. Fratiglioni et al. [67] found that a well-functioning social network is associated with a lower risk of cognitive deterioration. The effect of an engaging social network was also seen in this study—out of twenty-six participants in the IG, seven joined a dance club after the study to continue active group dancing.

### 4.1. Balance

The combination of the SOT and LOS provides comprehensive information about an individual’s ability to maintain balance under postural challenges. Measures of sensory integration, sway velocity, endpoint displacement, RT, and directional control are critical to identifying impairments that increase the ROF. The ability to control balance declines with age [68]. Such declines in balance performance are typically related to reduced sensory integration, such as vision, contrast sensitivity, depth perception, and visual flow, combined with a decline in neuromuscular function [5,6]. Furthermore, declines in balance performance can lead to an increased likelihood of falls, which can contribute to a loss of independence, especially if one suffers an injury or develops a fear of falling [6,13]. In this context, SOT composite scores below 38 have been shown to be particularly effective at distinguishing between individuals who have reported no falls in the last six months and those who have reported two or more falls [69].

In this study, however, no significant interaction effects concerning SOT were seen. Instead, the visual system score in the total sample showed a significant main effect of time (F = 12.445, *p* = 0.001, η^2^_p_ = 0.242 [large]). The dance training conducted in this study may have less directly addressed balance performance, which may explain the absence of statistically significant group difference [61]. Thus, it can be assumed that dance training alone is not sufficient to achieve targeted improvements in the visual system leading to measurable benefits of balance performance. To achieve such improvements, further multimodal intervention programs may consider integrating specific visual training components in addition to dance [59].

The LOS assesses dynamic balance and indirectly evaluates attention through the RT [70]. The subject must respond to the “Go” signal, determine the required speed and distance to reach the target, maintain this position briefly, and then return to the starting position. From a clinical perspective, individuals with MCI show declines in attention and are often unable to adequately perform more complex postural tasks when compared to healthy peers [71]. In this study, no significant improvements were found following the intervention.

A potential explanation for this absence of significant effects is the relatively limited specificity of our dance training intervention to postural control demands. While the dance training incorporated elements of coordination, rhythm, and dynamic movement, it may not have provided sufficiently large challenges to induce measurable improvements in specific sensory and motor systems responsible for maintaining static and dynamic balance. Specific balance training interventions, such as those based on perturbation-based exercises, unstable surfaces, or dual-task postural challenges, may induce more pronounced adaptations [68,72]. Moreover, the moderate exercise intensity might have further limited the neuromuscular stimulus necessary to elicit substantial improvements in postural control [73,74]. Following this line of interpretation, a more targeted training approach may provide a more effective means to improve balance performance in older adults with MCI, although future research is necessary to verify this assumption.

Because older adults often experience age-related sensory changes, slower RT, and impaired motor control, SOT and LOS tests are especially important for analyzing postural control and neuromuscular performance in the aging population. Studies on balance during walking show that visual input and visual disturbances significantly increase postural sway and gait variability [75,76]. Combining SOT and LOS tests makes it possible to obtain relevant data to help assess and interpret balance performance and, indirectly, fall risk [77]. Recent studies also emphasize that age-related reductions in muscle strength, particularly in the hip abductors and adductors, significantly impair postural control and lateral stability; muscle groups that play a central role in lateral movements such as turning and kicking [78]. Overall, these results underscore the importance of tailored interventions for older adults with MCI to effectively improve postural control to reduce the ROF.

### 4.2. Muscle Contraction Velocity

In contrast to the isolated consideration of Dm or Tc, in which each parameter indicates specific aspects such as muscle stiffness or the proportion of slow-twitch fibers, Vc offers a more comprehensive insight into muscle functionality [51]. Vc has been shown to be particularly sensitive to training effects and muscular adaptations; for example, Vc decreases after intense training sessions and correlates closely with the reduction in maximal voluntary force, making it a suitable parameter for monitoring muscle fatigue and recovery processes [79]. For athletes depending on rapid muscle contractions, like sprinters or jumpers, decreases in Vc are deemed to show a negative effect [80].

Additionally, studies show that older adults who experience falls exhibit lower leg power than non-fallers, with reduced leg power serving as an early indicator of balance impairments and fall risk [81,82]. Age-related declines in leg power are driven, among other factors, by a reduced Vc [83]. A decreased ability to activate muscles relevant for postural control, which can be indexed by changes in Vc, thus may serve as indicators of a higher fall risk [83].

The six-month dance intervention performed in this study did not significantly influence Vc. One possible explanation could be an insufficient exercise intensity to induce such neuromuscular changes. Perhaps the moderate exercise intensity of the dance training, which was conducted at an average percentage of 64% of the maximum heart rate, may not have provided a sufficiently high mechanical load to induce changes in muscle contractile properties detectable by tensiomyography. Studies have shown that more intense training interventions often result in significant muscular adaptations compared to less strenuous ones [74,84]. Furthermore, age-related decreases in muscle plasticity, especially in older adults with MCI, may have attenuated potential muscular responses to the moderate exercise intensity [85]. Aging is associated with a reduction in satellite cell activity, which is essential for the repair of muscle fibers, and an impaired muscle regeneration capacity, both of which could diminish the muscle’s ability to adapt structurally to exercise stimuli [85,86]. Given that the number of studies using tensiomyography to assess intervention-related changes in the neuromuscular systems of older adults is limited [87], further research in this direction is required before more robust conclusions can be drawn.

### 4.3. Fear of Falling and Number of Falls

Reduced postural control is a strong intrinsic predictor for fall risk [88,89]. Falling can have severe consequences for older adults, even without injury, because an increased fear of falling (i.e., lower balance confidence) can lead to a loss of independence and lower life-space mobility [90]. Falling is significantly associated with a fear of falling, calling for interventions targeted to reduce the fear of falling to reduce the risk of falling [91].

Based on the results of paired-samples *t*-tests in the IG, the fear of falling was significantly reduced following the intervention (*p* = 0.032 [small]), and the number of falls showed a trend in reduction in the number of falls (*p* = 0.088 [small]). Although there are no statistically significant group differences at post-test, such changes were absent in the CG. Even if we urge caution when interpreting our observation of a lower fear of falling in the IG, this observation is, at least partly, consistent with the findings of a systematic review of Sherrington et al. [24], in which 81 trials with a total of 19,684 participants were analyzed by comparing all types of exercise with control intervention regarding the prevention of falls. Compared with a control group, balance and functional exercises reduced the rate of falls by 24% and the number of people experiencing one or more falls by 13%. Multiple types of exercise (most commonly balance and functional exercises plus resistance exercises) probably reduced the rate of falls by 34% and the number of people experiencing one or more falls by 22%. Tai Chi may have reduced the rate of falls by 19% as well as reduced the number of people who experience falls by 20%. In a meta-analysis by Blanco-Rambo et al. [27] especially focused on dance training, a significant difference was found between dance interventions and the control groups in the general analysis of fall risk assessed by timed up-and-go, Berg Balance Scale, and one-leg stand tests, which serve as fall predictors, in favor of the intervention group. A common explanation for the positive effect of dance on fall risk-related measures is centered around the benefits of dance training for the motor control of complex motor tasks such as maintaining postural control and gait ability [89,92,93]. A better motor control may be associated with improved self-efficacy in balance, which, in turn, may lower the fear of falling in older adults [94,95].

### 4.4. Limitations

Several limitations should be considered when interpreting the findings of the current study. This study employed a per-protocol analysis, excluding participants who did not complete the post-tests. As a result, the findings should be interpreted with some caution, as this may limit the generalizability of the results to broader, real-world populations [96]. The methodology regarding the application of tensiomyography for detecting differences in neuromuscular functions in older adults is still in its infancy. Hence, these findings should be interpreted cautiously, and further research is needed to confirm our observations. Furthermore, although the moderate exercise intensity observed in this study (i.e., average of 64% of the maximum heart rate) can be sufficient to promote cardiovascular benefits, this intensity may have been too low to induce substantial neuromuscular adaptations. Additionally, it should be considered that the study procedures were influenced by the COVID-19 pandemic and its countermeasures, affecting not only the planned study duration but also the participant’s lifestyle behavior (e.g., level of regular physical activity and sedentary behavior) [97,98], and thus may have skewed the magnitude of the to-be-expected effects of the dance training on our set of outcomes. However, it is important to note that a previous study from the DiADEM project [99], investigating the regular physical activity levels of participants of this study and a cohort of healthy adults before pandemic restrictions using the German Physical Activity Questionnaire 50+ [100], did not observe significant differences concerning the regular physical activity levels [101]. Nonetheless, the circumstances during the COVID-19 pandemic make it difficult to compare our results to studies that were not conducted during a pandemic or a period surrounding such an event. More specifically, to adhere to COVID-19 pandemic regulations, the IG was divided into two separate training groups, and the planned training duration was reduced from twelve to six months. Presumably, a longer intervention duration would have led to more pronounced effects on fall-related measures such as balance performance, which is supported by the findings of a systematic review and network meta-analysis that recommends long-term maintenance of exercise programs to increase benefits of training interventions in older adults [54]. Thus, future research addressing those limitations is necessary to verify or refute our observations.

### 4.5. Suggestions for Further Research

To address the limitations of this study, we recommend that future studies increase the training duration and focus on smaller groups of participants to better tailor the dance training to the individual needs (e.g., capacity and performance level) of the participants, especially in vulnerable populations such as older adults with MCI. In addition to a longer training duration, integrating exercises into the dance choreographies that aim to improve balance performance (e.g., challenging dance step sequences including obstacles) may be a promising strategy to induce more pronounced effects in specific measures of postural control (i.e., dynamic and static balance).

## 5. Conclusions

In conclusion, our study did not provide evidence that six months of dance training in older adults with MCI would lead to statistically significant changes in postural or neuromuscular function (i.e., Vc), and number of falls. Fear of falling was significantly reduced in the IG; however, as the FES-I could not be analyzed using ANOVA, this result was only demonstrated through a paired-samples *t*-test. Our null findings are not fully consistent with previous evidence, which typically points towards positive effects of dance training on postural control and fall-related measures. Such differences between our study and previous studies are perhaps related to several factors, including, but not limited to, the relatively short intervention period—which was reduced due to COVID-19 restrictions—as well as an exercise intensity and specificity which were insufficient to induce measurable changes in our set of outcomes. Thus, future research is required to better understand how moderators such as training duration influence the effectiveness of dance training on postural control and fall-related measures among older adults with MCI.

## Figures and Tables

**Table 1:** General characteristics of the participants.

Characteristics	Total	Intervention Group	Control Group	*p*
Total (n)	50	26	24	-
Sex (female/male)	27/23	16/10	11/13	-
Subtype of MCI (amnestic/non-amnestic)	25/25	15/11	10/14	-
Age (years)	69.9 ± 6.2	70.7 ± 5.6	69.1 ± 6.8	0.373
Height (cm)	171.5 ± 9.2	169.1 ± 8.9	174.0 ± 9.0	0.060
Body weight (kg)	74.4 ± 11.3	71.8 ± 8.2	77.1 ± 13.5	0.097
Years of education	15.6 ± 2.5	15.8 ± 2.7	15.3 ± 2.1	0.396
Score MMSE	27.2 ± 1.4	27.1 ± 1.5	27.3 ± 1.2	0.651

Note: MCI = mild cognitive impairment; MMSE = mini-mental state examination.

**Table 2:** Analysis of the Sensory Organization Test.

	Control Group				Intervention Group				ANOVA		
	Pre		Post		Pre		Post				
	Mean ± SD	95% CI	Mean ± SD	95% CI	Mean ± SD	95% CI	Mean ± SD	95% CI	F	*p*	η^2^_p_
Composite Score	81.2 ± 3.9	[79.2, 83.1]	81.0 ± 3.3	[79.2, 82.8]	81.5 ± 4.5	[79.7, 83.4]	80.7 ± 4.2	[79.1, 82.4]	0.208	0.651	0.005
SOM	97.7 ± 1.1	[96.7, 98.6]	97.6 ± 1.8	[96.7, 98.6]	97.1 ± 2.7	[96.2, 98.0]	97.3 ± 2.2	[96.5, 98.2]	0.137	0.714	0.003
VIS	85.9 ± 8.1	[82.5, 89.3]	91.4 ± 4.3	[89.3, 93,4]	88.3 ± 6.7	[85.2, 91,5]	91.1 ± 4.6	[89.2, 93.0]	1.346	0.253	0.033
VEST	68.1 ± 11.1	[62.8, 73.3]	64.5 ± 10.1	[59.4, 69.6]	68.5 ± 11.6	[63.6, 73.4]	65.3 ± 11.7	[60.6, 70.0]	0.005	0.942	0
PREF	99.5 ± 6.0	[96.9, 102.1]	99.1 ± 7.4	[95.2, 102.9]	99.2 ± 5.2	[96.8, 101.6]	99.3 ± 9.0	[95.7, 102.9]	0.034	0.854	0.001

Note: SOM = somatosensory system score; VIS = visual system score; VEST = vestibular system score; PREF = visual preference sensory score; CI = confidence interval [lower bound, upper bound].

**Table 3:** Analysis of the Limits of Stability Test.

	Control Group				Intervention Group				ANOVA		
	Pre		Post		Pre		Post				
	Mean ± SD	95% CI	Mean ± SD	95% CI	Mean ± SD	95% CI	Mean ± SD	95% CI	F	*p*	η^2^_p_
Forward RT	1.4 ± 0.7	[1.1, 1.8]	0.9 ± 0.6	[0.5, 1.3]	1.3 ± 0.7	[1.0, 1.6]	1.2 ± 0.9	[0.8, 1.6]	1.377	0.249	0.038
Forward MVL	1.7 ± 1.3	[1.1, 2.2]	2.5 ± 1.6	[1.7, 3.3]	2.0 ± 0.8	[1.5, 2.5]	2.5 ± 1.4	[1.8, 3.2]	0.634	0.431	0.018
Forward EPE	50.1 ± 21.5	[40.4, 59.8]	61.1 ± 28.1	[48.1, 74.2]	47.8 ± 17.2	[39.3, 56.3]	54.4 ± 23.8	[43.1, 65.8]	0.172	0.681	0.005
Forward MXE	72.5 ± 13.8	[64.9, 80.1]	80.8 ± 15.1	[72.3, 89.2]	70.4 ± 15.7	[63.8, 77.0]	79.9 ± 17.8	[72.5, 87.3]	0.041	0.84	0.001
Forward DCL	90.3 ± 7.1	[86.7, 93.8]	87.3 ± 8.6	[82.6, 92.0]	85.4 ± 6.8	[82.3, 88.5]	87.8 ± 9.8	[83.7, 91.9]	2.357	0.134	0.063
Right RT	1.2 ± 0.4	[0.9, 1.4]	0.9 ± 0.4	[0.7, 1.1]	1.1 ± 0.5	[0.9, 1.3]	1.0 ± 0.5	[0.7, 1.2]	0.126	0.724	0.004
Right MVL	2.3 ± 0.8	[1.7, 2.9]	3.4 ± 1.7	[2.6, 4.2]	3.0 ± 1.4	[2.5, 3.6]	3.1 ± 1.5	[2.4, 3.8]	4.458	0.042 ^†^	0.113
Right EPE	68.8 ± 12.0	[63.8, 73.7]	67.4 ± 17.9	[58.8, 76.0]	70.4 ± 7.8	[66.1, 74.8]	67.0 ± 16.1	[59.5, 74.5]	0.131	0.719	0.004
Right MXE	78.6 ± 5.4	[74.5, 82.8]	82.7 ± 9.5	[77.2, 88.2]	80.4 ± 9.7	[76.8, 84.0]	78.3 ± 11.7	[73.5, 83.1]	3.567	0.067	0.092
Right DCL	87.3 ± 4.4	[84.4, 90.2]	84.1 ± 6.3	[77.4, 90.7]	84.5 ± 6.5	[82.0, 87.1]	79.2 ± 16.4	[73.4, 85.0]	0.204	0.655	0.006
Backward RT	0.9 ± 0.4	[0.7, 1.1]	0.7 ± 0.5	[0.5, 0.9]	0.9 ± 0.4	[0.7, 1.1]	0.6 ± 0.4	[0.4, 0.8]	0.393	0.535	0.011
Backward MVL	1.9 ± 0.9	[−0.2, 3.9]	2.1 ± 1.0	[1.5, 2.8]	3.2 ± 5.3	[1.4, 5.0]	2.2 ± 1.4	[1.6, 2.7]	0.76	0.389	0.021
Backward EPE	39.1 ± 9.5	[33.1, 45.2]	46.7 ± 12.1	[40.0, 53.4]	44.7 ± 13.5	[39.4, 49.9]	43.9 ± 14.0	[38.1, 49.7]	2.191	0.148	0.059
Backward MXE	72.4 ± 12.3	[64.6, 80.2]	70.1 ± 9.0	[62.7, 77.5]	70.4 ± 17.3	[63.6, 77.2]	62.5 ± 17.7	[56.0, 68.9]	0.822	0.371	0.023
Backward DCL	78.9 ± 8.4	[72.5, 85.3]	72.8 ± 14.8	[60.6, 85.0]	71.1 ± 15.0	[65.6, 76.7]	64.9 ± 29.1	[54.2, 75.5]	0.001	0.978	0
Left RT	1.0 ± 0.5	[0.8, 1.3]	0.9 ± 0.6	[0.5, 1.3]	0.9 ± 0.4	[0.7, 1.1]	1.0 ± 0.9	[0.6, 1.3]	0.494	0.487	0.014
Left MVL	2.8 ± 1.1	[2.2, 3.5]	3.4 ± 1.4	[2.7, 4.2]	3.1 ± 1.4	[2.6, 3.7]	3.3 ± 1.4	[2.7, 4.0]	0.696	0.41	0.019
Left EPE	73.4 ± 9.4	[67.0, 79.9]	72.3 ± 10.5	[64.7, 79.8]	64.7 ± 14.7	[59.1, 70.3]	70.1 ± 17.5	[63.5, 76.7]	1.253	0.271	0.035
Left MXE	81.4 ± 5.8	[75.4, 87.3]	81.5 ± 9.2	[76.8, 86.2]	78.40 ± 14.7	[73.2, 83.6]	81.8 ± 9.1	[77.7, 85.8]	0.617	0.438	0.017
Left DCL	87.8 ± 4.3	[85.0, 90.6]	86.4 ± 5.5	[83.5, 89.3]	85.7 ± 6.3	[83.3, 88.2]	85.4 ± 5.8	[82.9, 87.9]	0.167	0.685	0.005

Note: RT = reaction time (s); MVL = movement velocity (°/s); EPE = end point excursion (%); MXE = maximum excursion (%); DCL = directional control (%); CI = confidence interval [lower bound, upper bound]; ^†^ = the interaction effect did not remain significant following false discovery rate correction (Benjamini–Hochberg, Q = 0.05).

**Table 4:** Analysis of the muscle contraction velocity.

	Control Group				Intervention Group				ANOVA		
	Pre		Post		Pre		Post				
	Mean ± SD	95% CI	Mean ± SD	95% CI	Mean ± SD	95% CI	Mean ± SD	95% CI	F	*p*	η^2^_p_
Vc m. rectus femoris (left)	0.3 ± 0.1	[0.2, 0.3]	0.2 ± 0.1	[0.2, 0.3]	0.3 ± 0.1	[0.2, 0.3]	0.2 ± 0.9	[0.2, 0.2]	0.587	0.448	0.013
Vc m. rectus femoris (right)	0.3 ± 0.1	[0.3, 0.3]	0.3 ± 0.1	[0.2, 0.3]	0.3 ± 0.1	[0.2, 0.3]	0.2 ± 0.1	[0.2, 0.3]	0.117	0.734	0.003
Vc m. semitendinosus (left)	0.2 ± 0.1	[0.2, 0.3]	0.2 ± 0.1	[0.2, 0.3]	0.2 ± 0.1	[0.2, 0.3]	0.1 ± 0.1	[0.1, 0.2]	1.698	0.200	0.038
Vc m. semitendinosus (right)	0.2 ± 0.1	[0.2, 0.2]	0.2 ± 0.1	[0.1, 0.2]	0.2 ± 0.1	[0.1, 0.2]	0.1 ± 0.1	[0.1, 0.2]	0.498	0.484	0.011
Vc m. tibialis anterior (left)	0.1 ± 0.1	[0.1, 0.1]	0.1 ± 0.1	[0.1, 0.1]	0.1 ± 0.1	[0.1, 0.1]	0.1 ± 0.1	[0.1, 0.1]	0.133	0.717	0.003
Vc m. tibialis anterior (right)	0.1 ± 0.1	[0.1, 0.1]	0.1 ± 0.1	[0.1, 0.1]	0.1 ± 0.1	[0.1, 0.1]	0.1 ± 0.1	[0.1, 0.1]	0.02	0.889	0

Note: Vc = contraction velocity (mm/ms); CI = confidence interval [lower bound, upper bound].

**Table 5:** Analysis of the FES-I and number of falls.

	Control Group				Paired-Samples *t*-Test	Intervention Group				Paired-Samples *t*-Test
	Pre		Post			Pre		Post		
	Mean ± SD	95% CI	Mean ± SD	95% CI	*p*	Mean ± SD	95% CI	Mean ± SD	95% CI	*p*
Score	19.7 ± 4.3	[17.5, 21.8]	19.7 ± 4.9	[17.3, 22.1]	1	18.9 ± 2.5	[17.8, 20.0]	18.3 ± 2.9	[17.0, 19.5]	0.032 *
Number of Falls	0.7 ± 2.4	[−0.4, 1.9]	0.4 ± 1.3	[−0.2, 1.1]	0.593	0.4 ± 0.9	[0.0, 0.7]	0.00 ± 0.2	[0.0, 0.13]	0.088

Note: CI = confidence interval [lower bound, upper bound]; * displays significance.

## Data Availability

The raw data supporting the conclusions of this article will be made available by the authors on request.

## Abbreviations

The following abbreviations are used in this manuscript:

MCI	mild cognitive impairment
IG	intervention group
CG	control group
SOT	Sensory Organization Test
LOS	Limits of Stability Test
Vc	muscle contraction velocity
FES-I	Falls Efficacy Scale—International
ROF	risk of falling
RT	reaction time
MVL	movement velocity
COG	center of gravity
MXE	maximum excursion
EPE	endpoint excursion
DCL	directional control
ANOVA	analysis of variance
